# Deletion of Perilipin 5 Protects against Hepatic Injury in Nonalcoholic Fatty Liver Disease via Missing Inflammasome Activation

**DOI:** 10.3390/cells9061346

**Published:** 2020-05-28

**Authors:** Anastasia Asimakopoulou, Kathrin M. Engel, Nikolaus Gassler, Thilo Bracht, Barbara Sitek, Eva M. Buhl, Stavroula Kalampoka, Manuela Pinoé-Schmidt, Josef van Helden, Jürgen Schiller, Ralf Weiskirchen

**Affiliations:** 1 Institute of Molecular Pathobiochemistry, Experimental Gene Therapy and Clinical Chemistry (IFMPEGKC), RWTH University Hospital Aachen, 52074 Aachen, Germany; kalabokastivi@yahoo.gr (S.K.); manupisch@web.de (M.P.-S.); 2 Institute for Medical Physics and Biophysics, Leipzig University, Medical Faculty, 04107 Leipzig, Germany; Kathrin.Engel@medizin.uni-leipzig.de (K.M.E.); juergen.schiller@medizin.uni-leipzig.de (J.S.); 3 Section Pathology, Institute of Legal Medicine, University Hospital Jena, 07747 Jena, Germany; Nikolaus.Gassler@med.uni-jena.de; 4 Ruhr-University Bochum, Medical Faculty, Medizinisches Proteom-Center, 44801 Bochum, Germany; Thilo.Bracht@ruhr-uni-bochum.de (T.B.); barbara.sitek@ruhr-uni-bochum.de (B.S.); 5 Department of Anesthesia, Intensive Care Medicine, and Pain Therapy, University Hospital Knappschaftskrankenhaus Bochum, 44892 Bochum, Germany; 6 Electron Microscopy Facility, Institute of Pathology, RWTH Aachen University Hospital, 52074 Aachen, Germany; ebuhl@ukaachen.de; 7 Department of Biomedical and Biotechnological Sciences, University of Catania, 95100 Catania, Italy; 8 Laboratory Mönchengladbach—MVZ Dr. Stein and Colleagues, 41169 Mönchengladbach, Germany; JvHelden@labor-stein.de

**Keywords:** PLIN5, NAFLD, NASH, lipid droplets, NLRP3, inflammation, liver, hepatocytes, inflammasome, steatohepatitis

## Abstract

Nonalcoholic fatty liver disease (NAFLD) is a leading cause of chronic liver diseases with an increasing prevalence due to rising rates of obesity, metabolic syndrome and type II diabetes. Untreated NAFLD may progress to steatohepatitis (NASH) and ultimately liver cirrhosis. NAFLD is characterized by lipid accumulation, and when sufficient excess lipids are obtained, irreversible liver injury may follow. Perilipin 5 (PLIN5), a known lipid droplet coating protein and triglyceride metabolism regulator, is highly expressed in oxidatively modified tissues but it is still unclear how it affects NAFLD/NASH progress. We here studied how PLIN5 affects NAFLD development induced by a 30-week high-fat diet (HFD) administration in wild type and PLIN5 knock out (*Plin5*^−/−^) mice. The disruption of PLIN5 induced differences in lipid metabolism during HFD feeding and was associated with reduced hepatic fat accumulation. Surprisingly, *Plin5*^−/−^ mice showed mitigated activation of the NLR family pyrin domain-containing 3 (NLRP3) inflammasome, leading to minor hepatic damage. We conclude that PLIN5 is a pleiotropic regulator of hepatic homeostasis in NASH development. Targeting the PLIN5 expression appears critical for protecting the liver from inflammatory activation during chronic NAFLD.

## 1. Introduction

NAFLD has become one of the leading causes of chronic liver diseases in Western societies and is associated with obesity, insulin resistance, type II diabetes mellitus, hyperlipidaemia and metabolic syndrome [1]. The pathophysiology of NAFLD is quite complex. Despite the growing prevalence of NAFLD and its progress to NASH, liver fibrosis and ultimately cirrhosis and/or hepatocellular carcinoma, the underlying mechanisms of NAFLD are largely unknown. NAFLD is characterized mainly as simple steatosis present in more than 5% of hepatocytes [2]. Lipotoxicity during chronic untreated NAFLD endorses a ballooning degeneration of hepatocytes and inflammation. The histological establishment of fat accumulation, ballooned hepatocytes and inflammatory recruitment are essential conditions for the diagnosis and grading of NASH [3].

PLIN5 was identified independently by two groups as a member of the perilipin family that is expressed in highly oxidative tissues such as heart, liver and red muscle [4,5,6]. Its expression is induced during fasting conditions and regulated via the peroxisome proliferator activated-receptor α (PPAR-α) [4,5]. PLIN5 was given the role of an early responder to excess lipid intake in hepatocytes [7]. In a model of hepatic steatosis, *Plin5*^−/−^ mice showed defect lipid storage as well as increased lipolysis and fatty acid (FA) metabolism, leading to increased mitochondria proliferation. The hepatocytes of *Plin5*^−/−^ livers suffered from lipotoxic injury due to increased lipid peroxidation [8]. The same study revealed that PLIN5 disturbs adipose triglyceride lipase (ATGL)-mediated lipolysis by competitively binding to comparative gene identification-58 (CGI-58) and disrupting their interaction [8]. In a parallel study, Langhi et al. used statins to lower intracellular triglycerides (TG) and lipid droplets in primary hepatocytes, and found that statins reduce the expression of PLIN5 via the recruitment of the transcription factor sterol regulatory element-binding protein 2 (SREBP2), while PLIN5 overexpression reversed the effect of statins [9]. Moreover, while PLIN5 seems to be an important modulator of intrahepatic lipid metabolism and a key player in hepatosteatosis, *Plin5*^−/−^ mouse livers showed no differences in the expression of inflammatory markers during hepatosteatosis [10,11].

While hepatic PLIN5 has attracted significant interest regarding its role in lipid homeostasis, almost no information has been reported on the role of PLIN5 in the inflammatory pathogenesis of NAFLD/NASH. In a recent study, we found a relation of PLIN5 to inflammation in human liver samples obtained from patients suffering from hepatocellular carcinoma (HCC). In the respective study, we demonstrated that human HCC livers with exacerbated inflammation had a greatly higher *PLIN5* expression than non-HCC livers [12]. In this study, we observed that *Plin5*^−/−^ mice show mitigated liver injury and inflammasome activation, leading to a diminished inflammatory marker expression when challenged with a long-term HFD-induced NASH.

## 2. Materials and Methods

### 2.1. Generating Plin5-Deficient Mice

The *Plin5*^−/−^ mice were initially produced by using a standard gene disruption procedure with neo-cassette disrupting exons 2, 3 and 4, which are essential for the normal function of the PLIN5 gene [13]. Sperms from male *Plin5*^−/−^ mice were cryopreserved in 18% raffinose and 3% skimmed milk [14]. The *Plin5*^−/−^ mice strain was generated by insemination of C57BL/6J female mice with *Plin5*^−/−^ sperms. A straw with frozen spermatozoa was thawed from liquid nitrogen and warmed up at 37 °C for 15 min. The sperm solution was expelled to a plastic dish in a drop of 400 μL modified human tubal fluid medium (mHTF) for pre-incubation. After incubation at 37 °C and 5% CO_2_ for 45–60 min, the motility of sperm was checked and motile sperm from the periphery of the mHTF drop were then used for insemination. Heterozygous and homozygous offsprings for the disrupted genes were differentiated with conventional PCR with three oligos: the forward primer 5′-CACCCAGACCTGCTATAAGGACG-3′ with target location upstream of the neo-cassette, the reverse primer 5′-GAGAGTCAGCCCTGTGGAGTATTCTGG-3′, giving a product of 655 bp, and the reverse primer 5′-GCAGGTCGAGGGACCTAATAACTTCG-3′, targeting a location within the neo-cassette, giving a product of 305 bp.

### 2.2. Animals

C57BL/6 wild type (WT) and *Plin5*^−/−^ mice were housed 3–5 mice/cage. They were maintained at a constant temperature (20 °C) with a relative humidity of 50% and a 12 h of light and 12 h of darkness light cycle. WT (*n* = 24) and *Plin5*^−/−^ (*n* = 22) mice were fed ad libitum for 30 weeks on a mouse normal chow (NC) composed of 58% carbohydrates, 33% protein and 9% fat (V1534, ssniff Spezialdiäten GmbH, Soest, Germany), or a HFD containing 40% fat, 20% fructose and 2% cholesterol (D17010102, Research Diets, Inc., New Brunswick, NJ, USA). Food intake and body weight of the animals were monitored twice and once weekly, respectively. All animals used in this study received humane care, and all animal protocols were in full compliance with the guidelines for animal care approved by the institutional German Animal Care Committee (LANUV, Recklinghausen, Germany; permit no.: 84–02.04.2017.A268).

### 2.3. Liver Histology and NAFLD/NASH Scoring

At the end of week 30, all mice were fasted for 16 h prior to sacrifice to avoid postprandial effects. Water remained available ad libitum during fasting. Total body weight was measured and mice were sacrificed under inhalation anesthesia with isoflurane (Forene, Abbott, Wiesbaden, Germany). Blood was withdrawn from the inferior vena cava and livers were resected. After measuring liver weight, small tissue fragments were snap-frozen in liquid nitrogen and fixed in 4%-buffered paraformaldehyde, embedded in paraffin, and cut into 5 μm standard sections. Hematoxylin and eosin staining was performed using standard procedures and all sections were scanned with the NanoZoomer-SQ Digital slide scanner C13140-01 (Hamamatsu, Japan). For observation, the NDP.view2 viewer software (ver. 2.7.52) and the Nikon Eclipse E80 i light microscope (Nikon, Tokyo, Japan) were used. Histological scoring of murine steatosis, performed by a blinded pathologist (NG), was adapted from the guidelines used to score NAFLD/NASH in humans [15]. Liver sections were also kept for RNA or protein extraction as described previously [16].

### 2.4. Blood Analysis

Blood collected during sacrifice was split in the anticoagulant EDTA containing tubes for blood cell analysis as well as in gel containing silica serum tubes (both from Sarstedt, Nümbrecht, Germany). EDTA blood was used for the white blood cell count, while serum was used to measure alkaline phosphatase (AP), alanine aminotransferase (ALT), aspartate aminotransferase (AST), lactate dehydrogenase (LDH), triglycerides, glucose, lipase, cholesterol, bilirubin, iron, total protein and albumin. Routine hematological and biochemical parameters analysis were performed by blinded technicians. Blood serum was also kept frozen for Western blot analysis.

### 2.5. Isolation, Culture and Stimulation of Primary Hepatocytes

Hepatocytes were isolated from livers of 9∓13-week-old WT and *Plin5*^−/−^ male mice using the collagenase method [17]. Hepatocytes were cultured on collagen-coated dishes (3 × 10^4^ cells/well) in a Hepatozyme-SFM medium (Gibco, St. Louis, MO, USA). After cell adherence, the medium was changed to DMEM supplemented with 10% fetal calf serum (FCS), 1% pyruvate, 1% Penicillin/Streptomycin solution and 4 mM L-Glutamine (all from Sigma-Aldrich, Taufkirchen, Germany). The day after, the cells were starved for 3 h in DMEM containing 0.5% FCS and then stimulated with fatty acids or lipopolysaccharide (LPS) in a medium containing 0.2% FCS. After the respective treatments, the cells and supernatants were harvested for RNA and/or protein extraction as described elsewhere [16]. For stimulation experiments, primary hepatocytes from WT and *Plin5*^−/−^ mice cultured overnight (described above) were stimulated after starvation by the addition of 0.5 mM unsaturated oleic acid or saturated palmitic acid (both from Sigma-Aldrich). Untreated or vehicle-stimulated cells were used as the control. For LPS stimulation, primary hepatocytes were treated after the 3-h starvation period with 1 μg/mL LPS (Sigma-Aldrich) for 3 or 24 h.

### 2.6. RNA Expression Analysis and Statistical Analyses

Total RNA isolated from liver tissue or primary hepatocytes was extracted, purified and reverse-transcribed using standard protocols [18]. The oligo pairs used to amplify targeted mouse mRNAs via quantitative real time PCR were designed using the Universal Probe Library tool (Roche, Mannheim, Germany) and are listed in Table S1. The targeted mRNAs were amplified in 40 cycles and relative concentrations calculated using the comparative C_T_ method [19]. All mRNA quantities were normalized to the mRNA expression of either glyceraldehyde-3-phosphate dehydrogenase (Gapdh) or β-actin. The mRNA expression of treated mice or cells was finally normalized to the mRNA of the control groups. Statistical analyses of expression data were performed using the Student’s test for a comparison of the individual groups. Results are expressed as the mean ± standard deviation (SD). For in vitro experiments, the mean occurs from 3 independent trials each performed in triplicate. Probability values of ≤0.05 were considered statistically significant. All statistical analyses were performed using Excel Analysis 2010 and Windows 2010.

### 2.7. Western Blot Analysis

Total protein isolated from liver tissue and primary hepatocytes were prepared in RIPA lysis buffer as previously described [18]. Equal amounts of protein (20 μg/Lane for hepatocyte lysates and 100 μg/Lane for whole liver tissue extracts) were heated at 80 °C for 10 min before fractionation on 4–12% Bis-Tris gels in 2-(*N*-Morpholino)ethanesulfonic acid (MES) running buffer (Invitrogen, ThermoFisher Scientific, Schwerte, Germany). In the case of serum or cell supernatant, 5 µL and 45 µL, respectively, were mixed with Nu-PAGE™ LDS electrophoresis sample buffer containing dithiothreitol (DTT) and RIPA buffer before fractionation. Proteins were transferred via electroblotting onto a Protran membrane (Schleicher & Schuell, Dassel, Germany) for 2 h at 110 V. Equal protein loading was confirmed by Ponceau S staining. After electroblotting, the membranes were blocked by incubation with Tris-buffered saline (TBST) supplemented with 0.1% Tween 20 containing 5% (*w*/*v*) non-fat milk powder (both from Carl Roth, Karlsruhe, Germany) for 1 h at room temperature. Primary antibodies were applied overnight at 4 °C and visualized with anti-mouse, anti-rabbit, anti-goat (all from ThermoFisher Scientific) or anti-guinea pig IgG (Merck Millipore, Darmstadt, Germany) secondary antibodies and the SuperSignal chemiluminescent substrate (Pierce, Bonn, Germany) with the myECL TM Imager (ThermoFisher Scientific). All antibodies (Table S2) were diluted in 2.5% (*w*/*v*) non-fat milk powder in TBST prior to application to the membrane.

### 2.8. Electron Microscopy for Hepatic Mitochondria Imaging

Shortly after dissection, liver tissue was cut in small pieces (1–2 mm) and fixed in 3% glutaraldehyde in 1× PBS. Samples were washed in 0.1 M Soerensen’s phosphate buffer (Merck, Darmstadt, Germany), post-fixed in 1% Osmium tetroxide (OsO_4_) (Roth, Karlsruhe, Germany) in 17% sucrose buffer (Merck) and dehydrated by ascending ethanol series (30%, 50%, 70%, 90% and 100%) for 10 min each. The last step was repeated 3 times. Dehydrated specimens were incubated in propylene oxide (Serva) for 30 min, in a mixture of Epon resin (Serva) and propylene oxide (1:1) for 1 h and finally in pure Epon for 1 h. Samples were embedded in pure Epon. Epon polymerization was performed at 90 °C for 2 h. Ultrathin sections (70–100 nm) were cut with an ultramicrotome (Reichert Ultracut S, Leica, Wetzlar, Germany) using a diamond knife (Diatome Ltd., Nidau, Switzerland) and picked up on Cu/Rh g (HR23 Maxtaform, Plano GmbH, Wetzlar, Germany). Contrast was enhanced by staining with 0.5% uranyl acetate and 1% lead citrate (both from EMS GmbH, Munich, Germany). Samples were viewed at an acceleration voltage of 60 kV using a Zeiss Leo 906 (Carl Zeiss AG, Oberkochen, Germany) transmission electron microscope. Pictures were acquired in magnifications of 6000×, 10,000×, 27,000× and 60,000×.

### 2.9. Proteomics

(a) Sample preparation and tryptic digestion. The tissue samples were lysed and homogenized in sample buffer (30 mM Tris, 7 M urea, 2 M thiourea, 0.1% sodium deoxycholate, pH 8.5) and the protein concentrations were determined using a Bradford assay (Bio-Rad, Hercules, CA, USA). The samples were diluted to lower the urea concentration using 50 mM ammonium bicarbonate buffer (pH 7.8) and 4 µg protein per sample were reduced using 5 mM DTT (final concentration) for 30 min at 37 °C, followed by alkylation with final 15 mM iodacetamide for 30 min at room temperature in the dark. The samples were digested with trypsin (Serva Electrophoresis GmbH, Heidelberg, Germany) over night at 37 °C. Samples were acidified with a final concentration of 0.5% trifluoroacetic acid to quench the enzyme activity and precipitate sodium deoxycholate which was subsequently removed by centrifugation. The supernatants were dried in a vacuum centrifuge and re-dissolved in 0.1% trifluoroacetic acid. (b) Label-free LC-MS/MS analysis. The liquid chromatography-mass spectrometry (LC-MS/MS) analysis was performed using an Ultimate 3000 RSLCnano system (ThermoFisher Scientific Inc., Dreieich, Germany) coupled online to an Orbitrap Elite mass spectrometer (ThermoFisher Scientific). Next, 300 ng tryptic peptides were injected in a volume of 15 µL and pre-concentrated with 0.1% trifluoroacetic acid on a trap column for 7 min (Acclaim PepMap 100, 300 μm × 5 mm, C18, 5 μm, 100 Å; flow rate 30 μL/min). Subsequently, the peptides were separated on the analytical column (Acclaim PepMap RSLC, 75 μm × 50 cm, nano Viper, C18, 2 μm, 100 Å) by a gradient from 5% to 40% solvent B over 98 min (solvent A: 0.1% formic acid, solvent B: 0.1% formic acid, 84% acetonitrile; flow rate 400 nL/min; column oven temperature 60 °C). The instrument was operated in the data-dependent mode. Full scan mass spectra were acquired at a resolution of 60,000 at 400 *m*/*z* in the Orbitrap analyzer within a mass range of 350–2,000 *m*/*z*. The 20 most abundant precursors were selected for the MS/MS analysis and tandem mass spectra were acquired after peptide fragmentation by collision-induced dissociation. (c) Protein identification and quantification. Protein identification was performed using the Proteome Discoverer Software (ver. 1.4, ThermoFisher Scientific). The mass spectra were searched against the UniProtKB/Swiss-Prot database restricted to Homo sapiens (20,408 entries, Release 2018_10) using the Mascot search engine (ver. 2.5). The mass tolerance was set to 5 ppm for precursor ions and 0.4 Da for fragment ions. One tryptic missed cleavage was considered as well as chemical modifications of methionine (oxidation, dynamic) and cysteine (carbamidomethyl, static). The peptide validator implemented in Proteome Discoverer was used to estimate the peptide confidence and only peptides with a *q*-value < 0.01 were considered for analysis. Ion intensity-based label-free quantification was performed using Progenesis QI for proteomics (ver. 2.0.5387.52102, Nonlinear Dynamics, Newcastle upon Tyne, UK). The LC-MS/MS runs were aligned to one run automatically chosen by the software and a master list of features considering retention time and *m*/*z* was generated considering peptide ions with a minimum of three isotopic peaks and charge states +2, +3 and +4. The peptide spectrum matches from Proteome Discoverer were imported into the software and matched to the respective features. Normalized protein abundances were analyzed using an in-house written R script conducting ANOVA followed by Tukey′s post hoc test. The false discovery rate (FDR) was controlled using the Benjamini and Hochberg method [20]. The fold change was calculated using the mean abundances. Proteins that were quantified with a minimum of 2 unique peptides and passed the following thresholds were considered to be significantly differentially abundant: pFDR value ≤ 0.05, pTukeyHSD ≤ 0.05, FC ≥ 2 or ≤ −2. Our final comparison was based on the differences observed among WT and *Plin5*^−/−^ fed on NC and the differences among WT and *Plin5*^−/−^ fed on HFD. In Tables S4 and S5, all significant differences between WT and *Plin5*^−/−^ mice after NC and HFD are depicted.

### 2.10. Lipidomics

(a) Tissue homogenization and lipid extraction. Liver samples were mixed with 1 mL methanol and transferred into ball mill tubes (Precellys^®^ ceramic-kit, CKMIX, VWR, Radnor, PA, USA). Samples were homogenized using the tissue homogenizer Precellys^®^ 24 for 2 × 20 s at 5000 rpm and put on ice immediately. After the transfer of the homogenized samples into glass vials, ball mill tubes were washed with 0.5 mL methanol. Lipids were extracted by the addition of 1.5 mL chloroform and 1.5 mL water. Samples were mixed vigorously and centrifuged at room temperature at 2500 rpm for 10 min. The lower organic phase was withdrawn by a Hamilton syringe and lipid extraction was repeated once more with an additional volume of 1.5 mL chloroform to maximize the extraction yields. Organic phases were combined. Samples were stored at −20 °C until analysis. Prior to analyses, aliquots of the organic phases were evaporated to dryness. (b) High Performance Thin-Layer Chromatography. To overcome potential suppression effects that are known for mass spectrometric methods based on “soft” ionization, crude lipid extracts were separated by high-performance thin-layer chromatography (HPTLC). After separation into the individual lipid classes, the analysis of individual lipid fractions could be performed conveniently by means of electrospray ionization-ion trap mass spectrometry (ESI-IT MS). HPTLC and ESI-IT MS measurements were performed according to [21]. Briefly, 10 µL of crude lipid extracts was sprayed onto HPTLC silica gel 60 glass plates (Merck KGaA, Darmstadt, Germany). Plates were developed with chloroform/ethanol/water/trimethylamine (30:35:7:35, by vol.) as the mobile phase. Lipids were visualized by dipping the entire plate in primuline (Direct Yellow 59, Sigma-Aldrich, Taufkirchen, Germany) at a concentration of 50 mg/L in acetone/water (80:20, *v*/*v*). Lipids were detected as colored spots upon illumination with UV light (366 nm). Single lipid fractions were marked with a pencil and automatically eluted by a Plate Express™ TLC plate reader (Advion, Ithaca, NY, USA) with methanol as the solvent and subsequently analyzed by direct infusion into the ESI mass spectrometer. (c) Phospholipase A_2_ (PLA_2_) digestion. A digestion of lipids with the enzyme PLA_2_ helps to elucidate the fatty acid (FA) compositions at the *sn*-1 and the *sn*-2 position of the phospholipids (PLs) due to the stereospecificity of the enzyme which specifically cleaves the FA in the *sn*-2 position of PLs. Thus, lipids were digested with PLA_2_ according to the protocol given in [22]. Briefly, aliquots of the lipid extracts were evaporated to dryness, dissolved in 100 µL 1 mg/mL aqueous PLA_2_ (from bee venom, EC number 3.1.1.4, 1775 U/mg solid, Sigma-Aldrich) solution and incubated at 37 °C for 2 h under gentle shaking at 500 rpm using a Mixing Block (Biostep^®^, Jahnsdorf, Germany). Lipids were extracted as described above but only with 100 µL chloroform and methanol, respectively. After evaporation of the solvent, the lipid phases were dissolved in 30 µL chloroform. A 10 µL-aliquot was diluted 1:10 in methanol and subsequently analyzed by ESI-IT MS. (d) Electrospray ionization-ion trap mass spectrometry. ESI-IT MS was performed on an Amazon SL mass spectrometer (Bruker Daltonics GmbH, Bremen, Germany) by direct infusion using the following settings: spray voltage 4.5 kV, end plate offset 500 V, nebulizer gas 7.3 psi, drying gas (N2) 3 L/min, capillary temperature 180 °C, flow rate 3 μL/min, sheath gas (He) flow rate 25 a.u. The spectra were acquired in the positive ion mode to analyze the lysophosphatidylcholine (LPC) fraction and in the negative ion mode to analyze the FA fraction, both with enhanced resolution. (e) Software. For ESI-IT measurements and subsequent analysis of the mass spectra, the Bruker “Trap Control” and “Data Analysis” (vers. 4.1) software were used, respectively. Statistical analyses were performed using GraphPad Prism 8 (GraphPad Software, San Diego, CA, USA). Mean values and standard deviations were calculated. For the verification of significances, nonparametric and two-tailed *t*-tests were performed. Statistical significance was determined using the Holm-Sidak method, with α = 0.05. Significance was indicated by *p* ≤ 0.05. The graphs were created by the same software.

## 3. Results

### 3.1. Lack of Plin5 Alleviates Fatty Liver Injury

**Figure 1:**
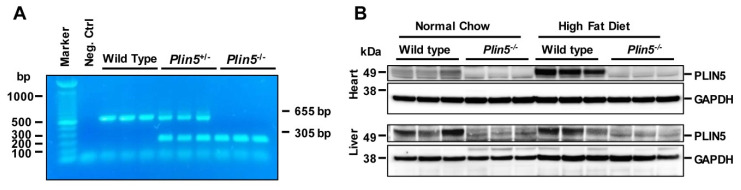
Generating of *Plin5*^−/−^ mice. (**A**) Confirmation of *Plin5* gene disruption by conventional PCR with genomic DNA prepared from mouse ear tips. Bands at 655 bp indicate wild type and 305 bp *Plin5* knockout alleles, while the presence of both bands indicates heterozygous mice. Neg. Ctrl = no template control. (**B**) Protein levels of PLIN5 revealed by Western blot analysis in hearts and livers of mice after 30 weeks on normal chow or high-fat diet. GAPDH served as a loading control.

The integrity of WT and *Plin5*^−/−^ heterozygous or homozygous litters used in our NAFLD studies were confirmed with conventional PCR on genomic DNA (Figure 1A) and Western blot analysis visualizing protein levels in liver and heart tissue (Figure 1B).

**Figure 2:**
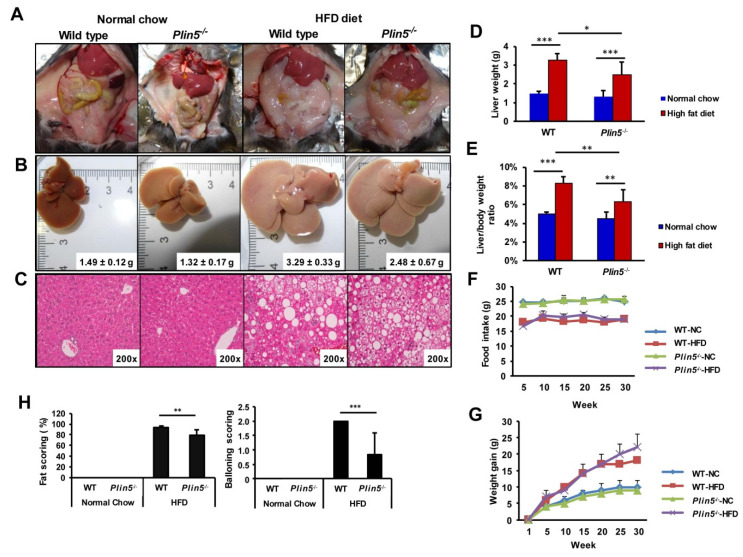
High-fat diet (HFD) induces less steatotic livers in *Plin5*^−/−^ mice. Wild type (WT) and *Plin5*^−/−^ mice fed either a normal chow or high-fat diet (HFD) for 30 weeks (*n* = 4–7 animals/group). (**A**) Overall image of the ventral area of representative mice after 30 weeks of diet, where organs such as liver, adipose tissues and intestines are visible. (**B**) Macroscopic appearance of WT and *Plin5*^−/−^ livers where HFD-induced hepatomegaly and a paler color on the livers due to fat accumulation with a lower effect on the *Plin5*^−/−^ livers are obvious. Average liver weight per group is indicated on the representative image. (**C**) Microscopic captions of liver sections’ histology stained with hematoxylin and eosin (200×). (**D**) Liver weight at time of sacrifice and (**E**) ratio of liver to body weight at the end of the experiment of all mice used. (**F**) Food intake and (**G**) weight gain per group monitored during the 30-week period. (**H**) Nonalcoholic fatty liver disease (NAFLD) scoring reveals less fat accumulation in the *Plin5*^−/−^ animals as well as fewer hepatocytes with ballooning degeneration after the HFD. Data are expressed as mean ± SD. Statistical analysis was performed with Student’s *t*-test. * *p* < 0.05; ** *p* < 0.01; *** *p* < 0.001.

WT and *Plin5*^−/−^ C57BL/6J mice were fed an HFD for 30 weeks (40% fat, 20% fructose and 2% cholesterol), while control mice received a NC (9% fat, 58% carbohydrates, 33% protein). All animals developed rich mesenterial adipose tissue as well as hepatomegaly after 30 weeks on the HFD, while WT mice had significantly higher liver weights, liver/body ratios and steatosis (Figure 2A–E). No differences were observed in food intake and weight gain between the genotypes (Figure 2F,G). NAFLD scoring from a blinded pathologist (NG) confirmed that all HFD fed mice had developed NAFLD with severe steatosis covering a 70–95% hepatic area. However, the *Plin5*^−/−^ mice accumulated significantly lower fat and showed lower amounts of ballooning degenerated hepatocytes (Figure 2H).

**Figure 3:**
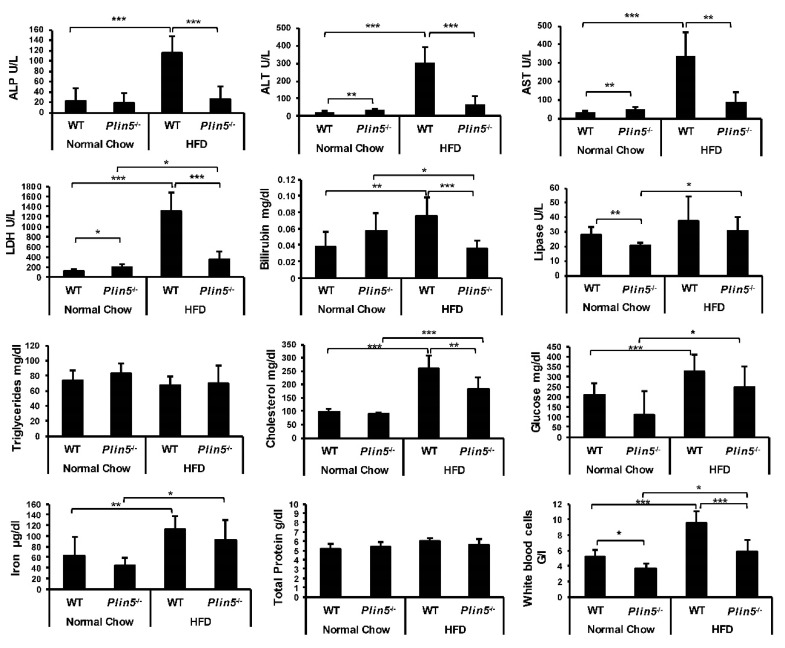
Absence of Perilipin 5 prevents high-fat diet (HFD)-induced liver injury. Serum and whole blood analysis from wild type (WT) and *Plin5*^−/−^ mice fed either a normal chow or HFD for 30 weeks (*n* = 4–7 animals/group). Serum analysis showed that enzymes such as alkaline phosphatase (ALP), aspartate aminotransferase (AST) and alanine transaminases (ALT) after HFD were only increased in WT. Lactate dehydrogenase (LDH), bilirubin, lipase, cholesterol, glucose, iron and white blood cells were differentially present among the different groups. Total protein and circulating triglycerides were present in all groups without statistical differences. Data are expressed as mean ± SD. Statistical analysis was performed with Student’s *t*-test. * *p* < 0.05; ** *p* < 0.01; *** *p* < 0.001.

In both WT and *Plin5*^−/−^ mice when fed an HFD, signs of important liver infiltration with lymphocytes, monocytes and plasma cells were absent. In addition, no neutrophils were detected (not shown). Moreover, detailed liver profile blood analyses revealed that concentrations of liver damage enzymes such as AP, AST and ALT were not increased in *Plin5*^−/−^ compared with WT mice (Figure 3).

The number of white blood cells was elevated after HFD feeding in both groups, with *Plin5*^−/−^ mice showing a statistically significant smaller increase. Bilirubin levels also indicated that WT mice were more prone to NAFLD damage than *Plin5*^−/−^ mice. Interestingly, LDH after HFD were only increased in WT mice, while circulating TG that reflect chylomicrons and VLDL were not affected by the HFD. On the contrary, blood glucose was increased in the HFD groups without differences in genotypes. Lipase levels remained almost unchanged, while iron levels increased in WT and *Plin5*^−/−^ mice equally during NAFLD development. As expected, circulating cholesterol levels increased after HFD feeding. However, these were significantly higher in WT mice.

### 3.2. Plin5 Regulates Mitochondrial State and ER-Stress

In the comparative LC-MS/MS proteomic analysis, we identified 71 proteins that were differentially expressed in WT animals under the different feeding conditions. In contrast, in *Plin5*^−/−^, only 33 proteins were identified showing a differential expression under the two feeding regimens. When comparing the two genotypes, we identified 17 differentially expressed proteins during NC feeding and 36 proteins during HFD feeding. However, none of these protein differences were present in both diets. As shown in Tables S3 and S4, *Plin5*^−/−^ mice fed on the NC showed an increased expression of ribosomal proteins and cytochrome p450 enzymes. The elevated cytochrome protein expression continued to be increased in the *Plin5*^−/−^ mice fed the HFD. Ribosomal and cytochrome p450 enzymes play a key role in the synthesis and metabolism of various molecules. They are located in the endoplasmatic reticulum (ER) and mitochondria involved in protein processing and transport.

**Figure 4:**
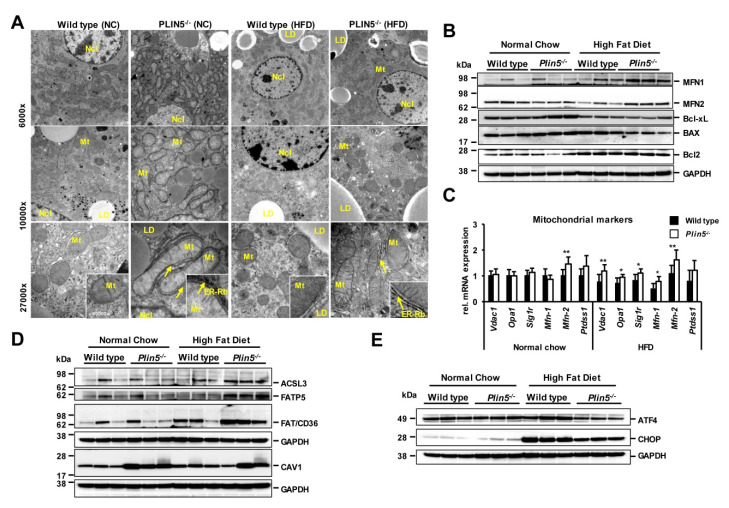
*Plin5* affects mitochondrial homeostasis. (**A**) Representative parenchymal liver pieces from wild type (WT) and *Plin5*^−/−^ animals fed a normal (NC) and high-fat diet (HFD) (*n* = 3/group) were observed after fixation with electron microscopy for hepatic mitochondria. Magnifications used were the 6,000×, 10,000×, 27,000× and 60,000×, respectively. The 60,000× magnification is given in a smaller square part of the 27,000× magnification images. Representative mitochondria (Mt), lipid droplets (LD) and nuclei (Ncl) are labeled and yellow arrows indicate the endoplasmic reticulum-ribosome presence (ER-Rb). (**B**) Western blot analysis of mitochondrial and apoptotic markers. (**C**) mRNA levels of mitochondrial markers involved in structure and function were tested in all experimental groups (*n* = 4–7 animals/group). Data are expressed as mean ± SD. Statistical analysis was performed with Student’s *t*-test. * *p* < 0.05; ** *p* < 0.01. (**D**,**E**) Western blot analysis of proteins related to fatty acid metabolism, endoplasmic reticulum (ER)-mitochondria and ER stress. Western blot depicting the results from 3 representative animals of each group.

To further test whether PLIN5 affects the mitochondria, we used electron microscopy to visualize hepatic mitochondria from the WT and *Plin5*^−/−^ mice after feeding NC or HFD for 30 weeks. The *Plin5*^−/−^ mice had enlarged mitochondria, covering most of the hepatocyte volume with a very intense presence of surrounding ER and ribosomes mostly when fed the NC but also after the HFD (Figure 4A). Therefore, several mitochondrial markers in RNA and protein levels were investigated. Markers related to apoptosis such as anti-apoptotic B-cell lymphoma-extra-large (Bcl-xL), B-cell lymphoma 2 (Bcl2) and pro-apoptotic Bcl2-associated X (BAX) were expressed equally in the two genotypes (Figure 4B). However, markers such as Mitofusin 1 (*Mfn-1*) and Mutofusin 2 (*Mfn-2*), encoding key proteins for mitochondrial fusion, showed significantly higher mRNA levels in the *Plin5*^−/−^ livers, which could probably explain the difference in mitochondrial size in the respective animals. Some other mitochondrial markers including optic atrophy 1 (*Opa1*), sigma-1 receptor (*Sig1r*) and voltage-dependent anion channel 1 (*Vdac1*) showed increased transcription in the knock out animals during HFD feeding, indicating that *Plin5* could participate in mitochondrial structure and trafficking (Figure 4C).

Next, the expressions of key mediators of FA metabolism related to ER and mitochondria such as long-chain acyl-CoA synthetase 3 (ACSL3), fatty acid translocase/cluster of differentiation 36 (FAT/CD36) and fatty acid transport protein 5 (FATP5) were analyzed (Figure 4D). ACSL3 is located in the ER and LDs and promotes lipogenesis and LD formation [23]. The *Plin5*^−/−^ animals exhibited an increase in FATP5 expression correlated with improved NAFLD histological progression [24]. The expression of CD36 was increased in the *Plin5*^−/−^ animals after HFD, and as shown before, correlates with a decreased autophagy in mouse livers [25]. Last, the expression of caveolin 1 (CAV1), a marker of lipid regulation and mitochondrial signaling which is known to have a protective role against hepatic steatosis and hepatocyte injury in NAFLD [26] was tested. *Plin5*^−/−^ livers had an increased expression of CAV1 under NC and HFD in comparison with WT, supporting our results for reduced steatosis and liver damage in those mice (Figure 4D).

In combination, the electron microscopy and proteomic analysis on *Plin5*^−/−^ livers confirmed induced mitochondrial markers and an ER–mitochondria interaction compared with WT livers. Transcription levels of numerous markers of lipolysis, lipogenesis and lipid coating were next tested. Markers for lipolysis such as *Atgl*, diacylglycerol lipase (*Dgl*) and monoacylglycerol lipase (*Mgl*) were higher expressed in the *Plin5*^−/−^ animals during NC feeding but the differences were alleviated after HFD administration (Figure S1A). *Plin2* and *Plin3* were induced in the *Plin5*^−/−^ animals, while *Plin4* followed the PLIN5 pattern. These differences were diminished after lipid overload (Figure S1B). Lipogenesis based on diacylglycerol acyltransferase 1 (*Dgat1*) and *Srebp1-α* were induced in *Plin5*^−/−^ animals after the HFD (Figure S1C). Moreover, ER stress induced by lipid overload was observed to be lower progressed in the *Plin5*^−/−^ mice. The protein activated transcription factor 4 (ATF4) is lower expressed in *Plin5*^−/−^ livers following HFD than in WT (Figure 4E). In stressful conditions, ATF4 is known to act together with DNA damage-inducible transcript 3 (CHOP) to induce apoptotic cell death [27]. In line with this assumption, the CHOP protein was found much less present in *Plin5*^−/−^ than in WT animals after HFD feeding, agreeing with the ballooning scoring indicating reduced hepatocyte death [28,29].

### 3.3. The Absence of Plin5 Mitigates the Pro-Inflammatory Response in Steatotic Livers

**Figure 5:**
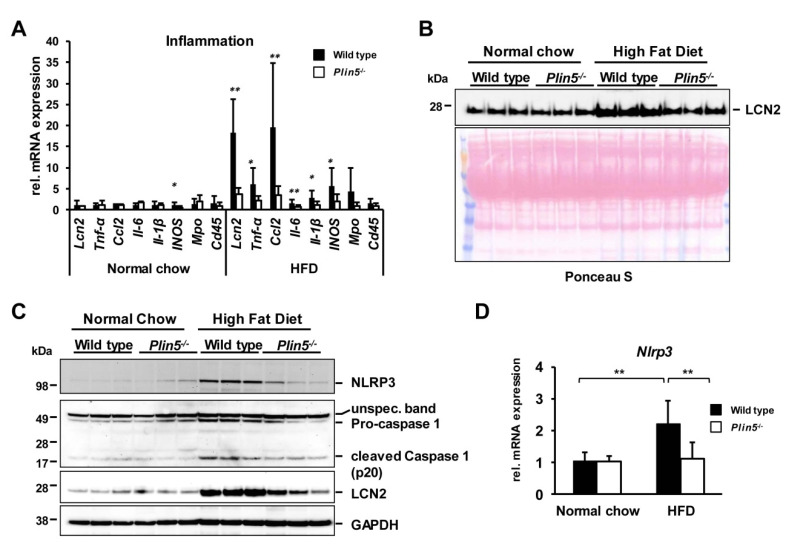
Suppression of *Plin5*-mitigated inflammation via missing activation of the NLRP3 inflammasome. (**A**) mRNA levels of major cytokines and inflammatory markers induced in the wild type (WT) animals after 30 weeks on the high-fat diet (HFD) (*n* = 4–7 animals/group). (**B**) Western blot of circulating pro-inflammatory marker Lipocalin 2 (LCN2) in serum of representative WT and *Plin5*^−/−^ mice after normal chow (NC) or HFD feeding. Ponceau S staining was used to demonstrate equal protein loading. (**C**) Western blot of protein levels of constituents of the inflammasome complex and LCN2 in WT and *Plin5*^−/−^ mouse livers after the NC or HFD administration. (**D**) *Nrlp3* mRNA expression in mouse livers. Data in (**A**) and (**D**) are expressed as mean ± SD. Statistical analysis was performed with Student’s *t*-test. * *p* < 0.05; ** *p* < 0.01.

Since NAFLD progression to NASH requires the development of inflammation, inflammatory marker gene transcription including the chemokine (C-C motif) ligand 2 (Ccl2), Lipocalin 2 (*Lcn2*), interleukin-6 (*Il-6*), *Tnf-α*, *Il-1β*, and inducible nitric oxide synthase (*Inos*) were analyzed (Figure 5A). Notably, all markers were increased after the HFD in WT animals but they presented almost no altered expression in the *Plin5*^−/−^ animals. Myeloperoxidase (*Mpo*) and the leukocyte common antigen 45 (*Cd45*) showed no significant upregulation in the HFD groups, confirming the pathologist observation for no leucocytes inflammatory cell recruitment in the hepatic tissue. The secreted pro-inflammatory marker LCN2 was found in the serum of all animals, with the highest concentrations in the serum of WT animals subjected to HFD using Ponceau S staining as the loading control (Figure 5B). In line, endogenous expression of LCN2 in hepatic extracts followed the same pattern as observed in serum (Figure 5C). Activation of the NLRP3 inflammasome has been recently demonstrated to play a crucial role in the progression of NASH [30]. Therefore, the expressions of the main complex proteins such as NLRP3 and Caspase 1 were investigated. NLRP3 was highest expressed after the HFD in the WT livers, which agrees with the *Nlrp3* transcription levels (Figure 5D). In the same line was the expression of the pro-Caspase 1, indicating an increased formation of the inflammasome complex in the WT animals that led further to the proteolytic cleavage activation of Caspase 1 (Figure 5C).

### 3.4. Lack of Plin5 Alleviates LPS-Induced Inflammasome Activation in Primary Hepatocytes by Suppressing NF-κB Signaling

**Figure 6:**
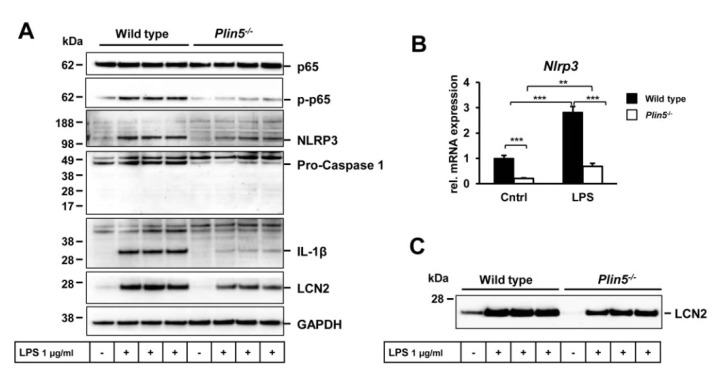
PLIN5 induces inflammasome activation via NF-κB in primary hepatocytes. (**A**) Western blot analysis of NF-κB (p65) and its activated phosphorylated form (*p*-p65) and markers of the inflammasome complex assembly and activation as well as the pro-inflammatory marker Lipocalin 2 (LCN2) in wild type (WT) and *Plin5*^−/−^ primary hepatocytes treated with lipopolysaccharide (LPS). Data shown are representative of 3 independent experiments done in triplicate for 24 h. (**B**) *Nrlp3* mRNA level in LPS-treated hepatocytes. Statistical analysis was performed with Student’s *t*-test. ** *p* < 0.01; *** *p* < 0.001. Data are shown as mean ± SEM of 3 independent experiments in triplicate. (**C**) Western blot of secreted LCN2 found in the supernatants of the WT and *Plin5*^−/−^ primary hepatocytes treated with LPS.

To understand further how PLIN5 affects inflammation via the inflammasome, primary hepatocytes from WT and *Plin5*^−/−^ animals were stimulated with LPS known to induce inflammasome activation [31]. The LPS endotoxic effect led to a strong activation of NF-κB (p65) in WT mice but only a slight one in the *Plin5*^−/−^ animals (Figure 6A). In line, the up-regulation of NLRP3, pro-Caspase 1 and the IL-1β precursor was alleviated in the *Plin5*^−/−^ hepatocytes (Figure 6A,B). In line with the animals’ serum findings, the pro-inflammatory protein LCN2 was found secreted in small amounts in the culture medium of the *Plin5*^−/−^ hepatocytes after the LPS stimulation (Figure 6C).

### 3.5. Lipid Profile Content of Plin5^−/−^ Livers Reveals Reduced Levels of the Inflammatory Mediator Arachidonic Acid

The lipid analysis by ESI-IT MS after the PLA_2_ digest revealed differences in the FA profile between the WT and *Plin5*^−/−^ livers (Table S5). Under the NC diet, there was more palmitic acid (C16:0) bound to the *sn*-2 position of PLs in the livers of the *Plin5*^−/−^ livers, while the linoleic acid (C18:2) showed no significant difference between the genotypes in the NC. A much lower level of arachidonic acid (C20:4) in the *Plin5*^−/−^ livers was observed. Considering the *sn*-1 position of PC molecules, there was also a decrease in C18:1, represented by LPC 18:1, in the *Plin5*^−/−^ livers in comparison with the WT animals. Challenging the mice with HFD modified the relative amounts of different FAs and LPCs between the different groups (Table S5).

**Figure 7:**
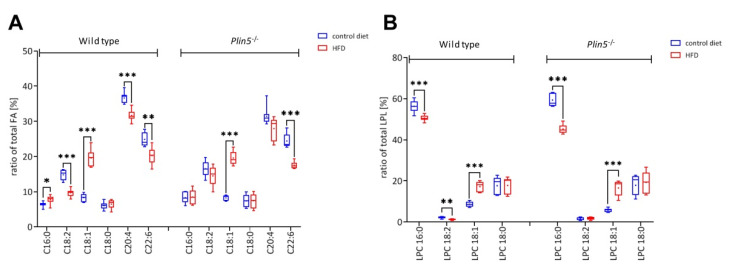
PLIN5 induces hepatic arachidonic acid. Lipid analysis by electrospray ionisation-ion trap mass spectrometry (ESI-IT MS) was performed in wild type (WT) and *Plin5*^−/−^ animals under normal chow (NC) and high-fat diet (HFD) 30-week administration (*n* = 4–7 animals/group). The phospholipase A_2_ enzymatic digest revealed (**A**) differences in the hepatic fatty acids cleaved from *sn*-1 position of phospholipids and (**B**) lysophosphatidylcholines (LPCs) including the head group with the glycerol back bone and the fatty acids in *sn*-2 position. Statistical analyses were performed using GraphPad Prism 8. Data are given as mean ± SD. For the verification of significances, nonparametric and two-tailed t-tests were performed. Statistical significance was determined using the Holm-Sidak method, with α = 0.05. Significance was indicated by * *p* < 0.05; ** *p* < 0.01; *** *p* < 0.001.

After 30 weeks on the HFD, a considerable increase in the relative amount of oleic acid (C18:1) bound to the *sn*-2 position of PLs in livers of the WT and *Plin5*^−/−^ mice was detected. The HFD increased palmitic acid (C16:0) in the WT animals only, closing the difference gap that they had under NC with the *Plin5*^−/−^ mice. This effect was accompanied by a reduction in linoleic acid (C18:2), with levels being significantly lower in the WT animals. Arachidonic acid (C20:4) was the only FA that insisted also after the HFD at a much lower level in the *Plin5*^−/−^ livers (Figure 7A, Table S5). In contrast to mice under the NC, LPC 16:0 levels were lower in *Plin5*^−/−^ mice fed a HFD compared with the WT animals. Levels of LPC 18:2 and LPC 18:1 behaved just like the respective FAs (Figure 7B).

## 4. Discussion

The pathophysiology of NASH is complex, including fat accumulation accompanied by inflammation and hepatocellular damage [1]. Pharmaco-therapeutic efforts to treat NAFLD include lipid-altering agents such as statins and nuclear receptor agonists that regulate multiple metabolic processes such as PPAR agonists used against hypertriglyceridemia [32,33]. When NAFLD progresses to NASH, in principal, drugs targeting inflammation and cell injury such as vitamin E improve liver histology in diabetic patients with NASH [33]. In NASH, PLIN5 has been suggested to respond to hepatosteatosis by regulating triglyceride metabolism, lipid storage, lipolysis and mitochondrial proliferation [7,8,9]. While inflammation is required for NASH development, PLIN5 has failed so far to show any relation to inflammation in models of hepatosteatosis [10,11].

In this study, we show that PLIN5 is a pleiotropic agent in hepatic homeostasis. Mice lacking PLIN5 showed attenuated liver injury in a HFD-induced NASH. First and unexpectedly, no alterations in the liver profile parameters in *Plin5*^−/−^ animals were observed after 30 weeks on an HFD, while the WT mice had developed a severe liver injury. While there were no differences in food intake and weight gain, we observed reduced hepatosteatosis in *Plin5*^−/−^ livers as shown before [8]. This was accompanied by a lower number of hepatocytes with ballooning degeneration. The lack of PLIN5 led to enlarged mitochondria with an intense presence of ER and ribosomes as well as an increased mitochondrial marker gene expression, facts that urge us to suggest that PLIN5 participates in mitochondrial homeostasis including structure and signaling. Moreover, FA transport was enhanced in the *Plin5*^−/−^ animals, which is in accordance with previous reports on the critical role of PLIN5 in mitochondrial capacity and oxidative metabolism [8,34]. Furthermore, ER stress-induced cell death signaling seemed to be suppressed in the knock out livers confirming the better hepatic state of these animals. This is in line with a reduced ER stress in *Plin5*^−/−^ animals which has already been reported in skeletal muscle [35].

The most pronounced effect in the presented NASH model was the mitigation of a pro-inflammatory response in the *Plin5*^−/−^ animals. A 30-week HFD induced NAFLD with the first signs of NASH (ballooned hepatocytes, monocyte infiltration) in this model. However, with the lack of leucocyte recruitment, particularly neutrophils, the establishment of NASH using routine histomorphological criteria cannot be diagnosed. Major monocyte chemoattractant- and pro-inflammatory cytokines were suppressed in the *Plin5*^−/−^ livers and in the circulation after the HFD. Since inflammasomes are known to be responsible for the activation of inflammatory responses, and particularly the NLRP3 inflammasome has a critical role in NAFLD development and its progress to NASH [30,36,37], the impact of PLIN5 on its activation was investigated. Surprisingly, the NLRP3 inflammasome activation was suppressed in the *Plin5*^−/−^ mice which consequently promoted hepatic inflammation. Targeting NLRP3 is a logical direction in pharmacotherapy of NASH and inhibiting its activation has been recently shown to be beneficial against inflammation and fibrosis in experimental NASH in mice [38]. The direct effect of PLIN5 on the activation of the NLRP3 inflammasome in primary hepatocytes via the well-known stimulant LPS [39,40] was shown. In this case, the *Plin5*^−/−^ hepatocytes blocked NF-κB (p65) signaling, leading to a suppressed activation of the inflammasome which reduced the hepatic expression of the precursor IL-1β. The significance of NF-κB in the NLRP3 inflammasome activation has already been reported before [38,41,42]. The final supportive fact in the regulation of inflammation by PLIN5 is the lipidomic analysis, which proved reduced levels of arachidonic acid in the *Plin5*^−/−^ mice. Arachidonic acid is considered an inflammatory mediator as it is metabolized to pro-inflammatory eicosanoids during an inflammatory response. Increased amounts of these metabolites have been reported during NASH progress [43].

## 5. Conclusions

In conclusion, this study shows that PLIN5 has an extended role as a regulator of liver homeostasis in NASH. Except for being a quick responder to lipid overload, PLIN5 has been proven to be essential for proper inflammatory signaling. Blocking the PLIN5 expression blocks the inflammatory response via the suppression of the NLRP3 inflammasome activation and improves the health status of mice with NASH. However, the lack of human samples in this study reduces its clinical importance. Whether blocking the *PLIN5* expression could be of therapeutic significance to human NASH patients needs to be further investigated.

## Supplementary Materials

The following are available online at https://www.mdpi.com/2073-4409/9/6/1346/s1, Figure S1: Hepatic lipid metabolism 30 weeks after normal chow and high-fat diet, Table S1: List of primers used in RT-qPCR, Table S2: List of primary and secondary antibodies used in Western blot analysis, Table S3: Differentially expressed proteins in liver extracts of wild type and *Plin5*^−/−^ mice that received a normal chow, Table S4: Proteomic analysis in high-fat diet samples, Table S5: Relative amounts of different fatty acids and lysophosphatidylcholines after phospholipase A_2_ digest.
