# Supplementary material for: Deletion of Perilipin 5 Protects against Hepatic Injury in Nonalcoholic Fatty Liver Disease via Missing Inflammasome Activation
Source: Cells. 2020 May 28;9(6):1346. doi: 10.3390/cells9061346 (PMC7348929; doi:10.3390/cells9061346)
Supplement: Supplementary file 1 [file cells-09-01346-s001.pdf]

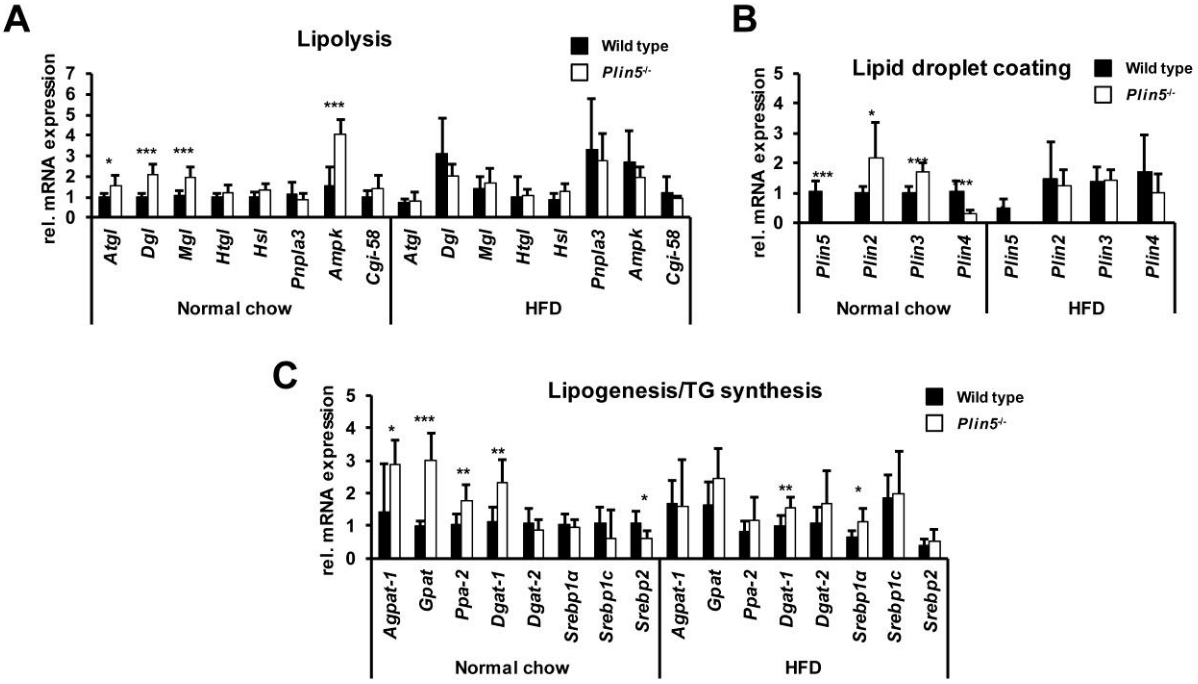

Figure S1: Hepatic lipid metabolism 30 weeks after normal chow and high fat diet (HFD). mRNA level of markers related to **(A)** lipolysis, **(B)** lipid droplet coating, and **(C)** lipogenesis from wild type and *Plin5* null mouse livers (n = 4-7 animals/group) were quantified and analyzed after the end of the experiment. Data of the mRNA levels are expressed as mean ± SD. Statistical analysis was performed with the Student's *t* test. **p* < 0.05; ***p* < 0.01; ****p* < 0.001. Abbreviations used are: *Agpat*-1, 1-acylglycerol-3-phosphate acyltransferase 1; *Ampk,* 5' AMP-activated protein kinase; *Atgl*, adipose triglyceride lipase; *Cgi-58,* comparative gene identification-58; *Dgat-1/2*, diacylglycerol acyltransferase 1/2; *Dgl,* diacylglycerol lipase; *Gpat,* Glycerol-3-phosphate acyltransferase; *Hsl*, hormone-sensitive lipase; HFD, high fat diet; *Htgl,* hepatic triglyceride lipase; *Mgl*, monoacylglycerol lipase; *Plin2/3/4/5*, perilipin 2/3/4/5; *Pnpla3,* Patatin-like phospholipase domain-containing protein 3; *Ppa*-2: inorganic pyrophosphatase 2; Srebp1α/1c/2, sterol regulatory element-binding protein 1α/1c/2; TG, triglycerides.

Table S1. List of primers used in RT-qPCR

Mouse Gene	Accession No./Reference	Forward primer (5'-3')	Reverse primer (5'-3')
β-actin	NM_007393	ctctagacttcgagcaggagatgg	atgccacaggattccatacccaaga
Agpat1	NM_018862.3	ctgtctgtggaagcaccttg	gcagaaccacagggtgga
Ampk	NM_001013367.3	ccttcgggaaagtgaaggt	gaatcttctgccggttgagt
Atgl	NM_001163689.1	tgaccatctgccttccaga	tgtaggtggcgcaagaca
Ccl2	NM_011333	gtgttggctcagccagatgc	gacacctgctgctggtgatcc
Cd45	NM_001111316.1	cttcagagaccacatatcatcca	gtctgcgagtcaggctgtg
Cgi-58	NM_026179.2	atctttggagcccgatcct	cttctggctgatctgcatacac
Dgat1	NM_010046.3	tttcagcaattatcgtggtatcc	aaaaataaccttgcattactcagga
Dgat2	NM_026384.3	gctggtgccctactccaag	ccagcttggggacagtga
Dgl	NM_198114.2	gagcaccaagcccaaatg	agctccgacttggggatac
Gapdh	XM_001473623	actgccacccagaagactg	caccaccctgttgctgtag
Gpat	NM_008149.3	ggaaggtgctgctattcctg	tgggatactggggttgaaaa
Hsl	NM_010719.5	agcgctggaggagtgtttt	ccgctctccagttgaacc
Htgl	AY228765.1	gacaaggcgtgggaacag	tttaatggcttgctagcttcagt
Il-1β	NM_008361	gagctgaaagctctccacctc	ctttcctttgaggcccaaggc
Il-6	NM_031168	gctaccaaactggatataatcagga	ccaggtagctatggtactccagaa
Inos	AY090567	ctttgccacggacgagac	tcattgtactctgagggctgac
Lcn2	NM_008491.1	ccatctatgagctacaagagaacaat	tctgatccagtagcgacagc
Mfn1	AY174062.1	gtgagcttcaccagtgcaaa	cacagtcgagcaaaagtagtgg
Mfn2	NM_001285920	cgaggctctggattcacttc	caaccagccagctttattcc
Mgl	NM_010719.5	tcggaacaagtcggaggt	tcagcagctgtatgccaaag
Mpo	NM_010824.2	gatggaatggggagaagctc	gcaggtagtcccggtatgtg
Nlrp3	NM_145827.4	tgctccctcttgtaccctgt	agaagagaccacggcagaag
Opa1	NM_001199177	ttcaccaggagaagtagactgtgt	tcttcaaataaacgcagaggtg
Pap-2	NM_008247.3	ggccctcgatgtgatttg	aatggatatactttgcccaatttt
Plin2	M93275.1	ctccactccactgtccacct-	gcttatcctgagcaccctga
Plin3	NM_025836.3	ccacaggatgctgaaaagg	tgatgtccctgaacatgctg
Plin4	NM_020568.3	ggacttacaaacagcaacagacc	tctgtgagttggtggacacttt
Plin5	NM_025874.3	gtccggtgatcagacagctc	tcgattcaccacattctgct
Pnpla3	NM_054088.3	tcccctcttctctggccta '	actcctccgtccacgtacc
Ptdss1	NM_008959.3	gttagcattccccaatggtc	ccaaaaaccattcgccata
Sig1r	NM_011014.3	gcaccacgaaaagtgaggtc	tttggtccccactccaga
Srebp1α	[1]	tagtccgaagccgggtgggcgccggcgccat	gatgtcgttcaaaaccgctgtgtgtccagttc
Srebp1c	[1]	atcggcgcggaagctgtcggggtagcgtc	actgtcttggttgttgatgagctggagcat
Srebp2	[1]	cacaatatcattgaaaagcgctaccggtcc	tttttctgattggccagcttcagcaccaatg
Tnf-α	NM_013693	accacgctcttctgtctactga	tccacttggtggtttgctacg
Vdac1	NM_011694.6	acctttgattcgtcattctcg	tgctccctcttgtaccctgt

[1] Shimomura, I.; Shimano, H.; Horton, J.D.; Goldstein, J.L.; Brown, M.S. Differential expression of exons 1a and 1c in mRNAs for sterol regulatory element binding protein-1 in human and mouse organs and cultured cells. *J. Clin. Invest.* **1997**, *99*, 838-845, doi: 10.1172/JCI119247.

Table S2. List of primary and secondary antibodies used in Western blot analysis

Primary antibodies					
Antibody	Cat. No.	Clonality	Supplier	Host	Dilution
ACSL3	sc-166374	Monoclon al	Santa Cruz	Mouse	1:500
ATF4	sc-390063	Polyclonal	Santa Cruz	Rabbit	1:1000
BAX	2772	Polyclonal	Cell Signaling	Rabbit	1:1000
BCL-2	2870	Polyclonal	Cell Signaling	Rabbit	1:1000
Bcl-xL	2764	Monoclon al	Cell Signaling	Rabbit	1:1000
Caspase 1	sc-514	Polyclonal	Santa Cruz	Rabbit	1:200
Caveolin 1	3238	Polyclonal	Cell Signaling	Rabbit	1:1000
CHOP	5554	Polyclonal	Cell Signaling	Rabbit	1:1000
FAT/CD36	PA5-27236	Polyclonal	ThermoFisher Scientific	Rabbit	1:500
FATP5	sc-377374	Monoclon al	Santa Cruz	Mouse	1:500
GAPDH	sc-32233	Monoclon al	Santa Cruz	Mouse	1:10,000
LCN2	AF1857	Polyclonal	R&D Systems	Goat	1:1000
MFN1	sc-166644	Monoclon al	Santa Cruz	Mouse	1:500
MFN2	sc-100560	Monoclon al	Santa Cuz	Mouse	1:500
NF-κB (p65)	sc-8008	Monoclon al	Santa Cruz	Mouse	1:1000
NF-κB (p-p65)	3033	Polyclonal	Cell Signaling	Rabbit	1:1000
NLRP3	15101	Polyclonal	Cell Signaling	Rabbit	1:500
PLIN5	GP31	Polyclonal	PROGEN	Guinea pig	1:1000
**Secondary antibodies**					
Antibody	Cat. No.	Clonality	Supplier	Species	Dilution
Goat anti-guinea pig IgG, HRP	AP108P	Polyclonal	Merck Millipore	Guinea pig	1:5000
Goat anti-mouse IgG (H+L), HRP	31430	Polyclonal	ThermoFisher Scientific	Mouse	1:5000
Goat anti-rabbit IgG (H+L, HRP	31460	Polyclonal	ThermoFisher Scientific	Rabbit	1:5000
Mouse anti-goat IgG (H+L), HRP	31400	Polyclonal	ThermoFisher Scientific	Goat	1:5000

Table S3. Differentially expressed proteins in liver extracts of wild type and *Plin5*$^{-/-}$ mice that received a normal chow

Accession	Description	p-value (*Plin5*$^{-/-}$/WT)	Ratios (*Plin5*$^{-/-}$/WT)
P43883	Perilipin-2 OS=Mus musculus GN=Plin2 PE=2 SV=2 - [PLIN2_MOUSE]	***	4.50
P09528	Ferritin heavy chain OS=Mus musculus GN=Fth1 PE=1 SV=2 - [FRIH_MOUSE]	***	-7.63
P29391	Ferritin light chain 1 OS=Mus musculus GN=Ftl1 PE=1 SV=2 - [FRIL1_MOUSE]	***	-2.99
P35980	60S ribosomal protein L18 OS=Mus musculus GN=Rpl18 PE=2 SV=3 - [RL18_MOUSE]	***	2.15
O88833	Cytochrome P450 4A10 OS=Mus musculus GN=Cyp4a10 PE=2 SV=2 - [CP4AA_MOUSE]	***	5.31
Q6ZWN5	40S ribosomal protein S9 OS=Mus musculus GN=Rps9 PE=2 SV=3 - [RS9_MOUSE]	***	2.15
O35728	Cytochrome P450 4A14 OS=Mus musculus GN=Cyp4a14 PE=2 SV=1 - [CP4AE_MOUSE]	***	6.30
P62301	40S ribosomal protein S13 OS=Mus musculus GN=Rps13 PE=1 SV=2 - [RS13_MOUSE]	***	2.05
P14148	60S ribosomal protein L7 OS=Mus musculus GN=Rpl7 PE=2 SV=2 - [RL7_MOUSE]	**	2.14
P12970	60S ribosomal protein L7a OS=Mus musculus GN=Rpl7a PE=2 SV=2 - [RL7A_MOUSE]	***	2.32
P47911	60S ribosomal protein L6 OS=Mus musculus GN=Rpl6 PE=1 SV=3 - [RL6_MOUSE]	***	2.56
Q64FW2	All-trans-retinol 13,14-reductase OS=Mus musculus GN=Retsat PE=1 SV=3 - [RETST_MOUSE]	***	2.13
Q6ZWV7	60S ribosomal protein L35 OS=Mus musculus GN=Rpl35 PE=2 SV=1 - [RL35_MOUSE]	***	2.38
Q9CR57	60S ribosomal protein L14 OS=Mus musculus GN=Rpl14 PE=2 SV=3 - [RL14_MOUSE]	***	2.96
P47915	60S ribosomal protein L29 OS=Mus musculus GN=Rpl29 PE=2 SV=2 - [RL29_MOUSE]	**	2.20
P43277	Histone H1.3 OS=Mus musculus GN=Hist1h1d PE=1 SV=2 - [H13_MOUSE]	***	3.62
A2ATU0	Probable 2-oxoglutarate dehydrogenase E1 component DHKTD1, mitochondrial OS=Mus musculus GN=Dhtkd1 PE=2 SV=1 - [DHTK1_MOUSE]	*	2.02

LC-MS/MS analysis was performed of wild type (WT) and *Plin5*$^{-/-}$ mouse livers fed on a normal chow for 30 weeks (n = 4-7 animals/group). Depicted are proteins identified by a minimum of two unique peptides that had differential expression between the two genotypes. Data are given as ratio of values for the *Plin5*$^{-/-}$ mouse livers to the WT ones. Only significant differences with pairwise $p < 0.05$ are given. * $p < 0.05$, ** $p < 0.01$, *** $p < 0.001$.

Table S4. Proteomic analysis in high fat diet samples

Accession	Description	p-value (*Plin5*$^{-/-}$/WT)	Ratio (*Plin5*$^{-/-}$/WT)
Q9QXZ6	Solute carrier organic anion transporter family member 1A1 OS=Mus musculus GN=Slco1a1 PE=2 SV=1 - [SO1A1_MOUSE]	***	3.81
Q9QXF8	Glycine N-methyltransferase OS=Mus musculus GN=Gnmt PE=1 SV=3 - [GNMT_MOUSE]	***	2.12
Q3TW96	UDP-N-acetylhexosamine pyrophosphorylase-like protein 1 OS=Mus musculus GN=Uap1l1 PE=2 SV=1 - [UAP1L_MOUSE]	***	-3.10
Q61694	3 beta-hydroxysteroid dehydrogenase type 5 OS=Mus musculus GN=Hsd3b5 PE=1 SV=4 - [3BHS5_MOUSE]	*	9.27
Q63836	Selenium-binding protein 2 OS=Mus musculus GN=Selenbp2 PE=1 SV=2 - [SBP2_MOUSE]	**	4.50
P56654	Cytochrome P450 2C37 OS=Mus musculus GN=Cyp2c37 PE=2 SV=2 - [CP237_MOUSE]	**	2.65
Q6XVG2	Cytochrome P450 2C54 OS=Mus musculus GN=Cyp2c54 PE=2 SV=1 - [CP254_MOUSE]	***	3.18
P19324	Serpin H1 OS=Mus musculus GN=Serpinh1 PE=1 SV=3 - [SERPH_MOUSE]	***	-2.27
P04939	Major urinary protein 3 OS=Mus musculus GN=Mup3 PE=1 SV=1 - [MUP3_MOUSE]	***	2.64
Q61599	Rho GDP-dissociation inhibitor 2 OS=Mus musculus GN=Arhgdib PE=1 SV=3 - [GDIR2_MOUSE]	***	-2.67
Q91X77	Cytochrome P450 2C50 OS=Mus musculus GN=Cyp2c50 PE=1 SV=2 - [CY250_MOUSE]	***	2.45
P28653	Biglycan OS=Mus musculus GN=Bgn PE=2 SV=1 - [PGS1_MOUSE]	***	-7.21
Q91WL5	Cytochrome P450 4A12A OS=Mus musculus GN=Cyp4a12a PE=2 SV=2 - [CP4CA_MOUSE]	***	2.89
B5X0G2	Major urinary protein 17 OS=Mus musculus GN=Mup17 PE=2 SV=2 - [MUP17_MOUSE]	**	5.328
P07356	Annexin A2 OS=Mus musculus GN=Anxa2 PE=1 SV=2 - [ANXA2_MOUSE]	*	-2.08
Q00915	Retinol-binding protein 1 OS=Mus musculus GN=Rbp1 PE=2 SV=2 - [RET1_MOUSE]	***	-3.13
Q9JK53	Prolargin OS=Mus musculus GN=Prelp PE=1 SV=2 - [PRELP_MOUSE]	***	-5.66
P17047	Lysosome-associated membrane glycoprotein 2 OS=Mus musculus GN=Lamp2 PE=2 SV=2 - [LAMP2_MOUSE]	*	2.01
Q8R0W0	Epiplakin OS=Mus musculus GN=Eppk1 PE=1 SV=2 - [EPIPL_MOUSE]	***	-2.55
P11589	Major urinary protein 2 OS=Mus musculus GN=Mup2 PE=1 SV=1 - [MUP2_MOUSE]	*	2.01
Q8BUV3	Gephyrin OS=Mus musculus GN=Gphn PE=1 SV=2 - [GEPH_MOUSE]	**	2.03
P01868	Ig gamma-1 chain C region secreted form OS=Mus musculus GN=Ighg1 PE=1 SV=1 - [IGHG1_MOUSE]	*	-3.03
P51885	Lumican OS=Mus musculus GN=Lum PE=1 SV=2 - [LUM_MOUSE]	**	-4.59

LC-MS/MS analysis of wild type (WT) and *Plin5*$^{-/-}$ mouse livers fed on a high fat diet for 30 weeks (n = 4-7 animals/group). Depicted are proteins identified by a minimum of two unique peptides that had differential expression between the two genotypes. Data are given as ratio of values for the *Plin5*$^{-/-}$ mouse livers to the WT ones. Only significant differences with pairwise $p < 0.05$ are given. *$p < 0.05$, ** $p < 0.01$, *** $p < 0.001$.

Table S5. Relative amounts of different fatty acids and lysophosphatidylcholines after phospholipase A_2 digest

**Normal chow**			**High fat diet**		
FA	WT	*Plin5*$^{-/-}$	FA	WT	*Plin5*$^{-/-}$
Pal C16:0	6.4 ± 0.7	8.4 ± 1.5**	C16:0	7.8 ± 1.2	8.5 ± 2.0
Lin C18:2	14.8 ± 1.4	16.5 ± 2.1	C18:2	9.7 ± 1.0	14.5 ± 2.7**
Ol C18:1	8.3 ± 1.1	8.2 ± 0.8	C18:1	19.6 ± 2.5	19.6 ± 1.9
C18:0	6.0 ± 0.9	7.4 ± 1.8	C18:0	6.5 ± 1.2	7.4 ± 2.1
Arac C20:4	36.6 ± 1.6	31.4 ± 2.4***	C20:4	31.7 ± 1.5	27.9 ± 3.1*
C22:6	24.9 ± 1.9	24.4 ± 1.9	C22:6	20.3 ± 2.4	17.5 ± 1.0**
LPC	WT	*Plin5*$^{-/-}$	LPC	WT	*Plin5*$^{-/-}$
LPC 16:0	56.4 ± 3.8	59.3 ± 3.4	LPC16:0	49.6 ± 2.8	45.1 ± 2.8**
LPC 18:2	2.3 ± 0.8	1.6 ± 0.8	LPC18:2	1.3 ± 0.5	1.7 ± 0.9
LPC 18:1	8.6 ± 1.5	5.8 ± 1.1***	LPC18:1	17.2 ± 2.7	16.4 ± 4.3
LPC 18:0	17.9 ± 4.7	17.1 ± 5.0	LPC18:0	17.7 ± 4.1	19.7 ± 6.4

Organic fractions of liver samples from wild type (WT) and *Plin5*$^{-/-}$ mice, fed either a normal chow or a high fat diet, were digested with PLA_2. This digestion results in fatty acids (FA) formerly bound to the *sn*-2 position of phospholipids and the lysophospholipids with one residual FA in the *sn*-1 position. Data are given as mean ± SD in % of total FA and total lysophosphatidylcholines, respectively. Significant differences between WT and *Plin5*$^{-/-}$ mice were determined using the Holm-Sidak method with $\alpha = 0.05$. Each row was analyzed individually, without assuming a consistent SD. The number of t tests was 12 for the FAs and 8 for LPCs. Significances are given as *$p < 0.05$, ** $p < 0.01$, *** $p < 0.001$.
